# Q-Elastography in the Presurgical Diagnosis of Thyroid Nodules with Indeterminate Cytology

**DOI:** 10.1371/journal.pone.0050725

**Published:** 2012-11-29

**Authors:** Vito Cantisani, Salvatore Ulisse, Eleonora Guaitoli, Corrado De Vito, Riccardo Caruso, Renzo Mocini, Vito D’Andrea, Valeria Ascoli, Alfredo Antonaci, Carlo Catalano, Francesco Nardi, Adriano Redler, Paolo Ricci, Enrico De Antoni, Salvatore Sorrenti

**Affiliations:** 1 Department of Radiology, Oncology and Antomy Pathology, “Sapienza” University of Rome, Rome, Italy; 2 Department of Experimental Medicine, “Sapienza” University of Rome, Rome, Italy; 3 Department of Surgical Sciences, “Sapienza” University of Rome, Rome, Italy; 4 Department of Public Health and Infectious Diseases, “Sapienza” University of Rome, Rome, Italy; Ospedale Pediatrico Bambino Gesù, Italy

## Abstract

Quantitative ultrasound (US) elastography (Q-USE), able to evaluate tissue stiffness has been indicated as a new diagnostic tool to differentiate benign from malignant thyroid lesions. Aim of this prospective study, conducted at the Department of Surgical Sciences, of the “Sapienza” University of Rome, was to evaluate the diagnostic accuracy of Q-USE, compared with US parameters, in thyroid nodules with indeterminate cytology (Thy3).The case study included 140 nodules from 140 consecutive patients. Patient’s thyroid nodules were evaluated by Q-USE, measuring the strain ratio (SR) of stiffness between nodular and surrounding normal thyroid tissue, and conventional US parameters prior fine-needle aspiration cytology. Those with Thy3 diagnosis were included in the study. Forty of the nodules analyzed harbored a malignant lesion. Q-USE demonstrated that malignant nodules have a significant higher stiffness with respect to benign one and an optimun SR cut-off value of 2.05 was individuated following ROC analysis. Univariate analysis showed that hypoechogenicity, irregular margins and SR >2.05 associated with malignancy, with an accuracy of 67.2%, 81,0% and 89.8%, respectively. Data were unaffected by nodule size or thyroiditis. These findings were confirmed in multivariate analysis demonstrating a significant association of the SR and the irregular margins with thyroid nodule’s malignancy. In conclusion, we demonstrated the diagnostic utility of Q-USE in the differential diagnosis of thyroid nodules with indeterminate cytology that, if confirmed, could be of major clinical utility in patients’ presurgical selection.

## Introduction

Despite being very common in the general population, only a minority of thyroid nodules harbor a malignant lesion [1]–[4]. Therefore, the first aim in their evaluation is to exclude malignancy [1]–[4]. In that regard, patient’s clinical data, ecographic parameters and other imaging procedures (i.e. FDG-PET and ^99 m^Tc-MIBI) or the search for genetic alterations (i.e. BRAF mutations and RET-PTC rearrangement) have been proved to be poor predictors of malignancy [5]–[12]. Fine-needle aspiration cytology (FNAC) represents the main diagnostic tool in the evaluation of palpable and not palpable thyroid nodules because of its high accuracy (84–95%), reproducibility and low cost [13]–[19]. However, FNAC is characterized by a grey diagnostic area (Thy3) due to the finding, in about 10% of the specimens, of cellular atypia of indeterminate significance which precludes a distinction between benign and malignant lesions [20]. As a consequence, about 80% of patients with Thy3 cytology undergo unnecessary thyroidectomy for the histopathological diagnosis [5], [13].

Over the last few years, the newly developed qualitative real-time ultrasound elastography (USE), which is capable of evaluating tissue stiffness by measuring the amount of distortion that occurs when the nodules are subject to external pressure, has been indicated as a new diagnostic tool for differentiating benign from malignant thyroid lesions [21]–[40]. A meta-analysis published in 2010 showed, in fact, that USE has a mean sensitivity and specificity of 92% and 95%, respectively, capable of improving FNAC accuracy and consequently restricting the number of patients recommended for surgery [23]. However, more recent reports do not confirm the usefulness of USE in the pre-surgical selection of nodules and suggest the need for a quantitative (Q-USE) assessment of nodule stiffness to improve USE diagnostic accuracy [35], [38], [39]. In fact, recent advances in elastography allow quantification using the strain ratio calculated as the ratio of stiffness between nodular tissue versus surrounding normal thyroid [22], [26].

This study, which includes 140 patients being operated following Thy3 cytological diagnosis, evaluates the diagnostic accuracy of Q-USE, compared with conventional US parameters.

## Patients and Methods

### Patients

The case study included 140 nodules from 140 consecutive patients (12 males and 128 females) with a median age of 38 yr (range 17–78 yr), enrolled between February 2009 and December 2011. Patients were admitted to the Department of Surgical Sciences, “Sapienza” University Rome, following a Thy 3 cytological diagnosis. The exclusion criteria included: presence of cystic part 10% greater than the nodule volume; nodules with egg shell calcification; nodules occupying more than 80% of the lobe and, finally, nodules located in the isthmus. All patients were in euthroidism or rarely in subclinical hypothyroidism as judged by serum TSH, FT_4_ and FT_3_ levels and none of them had undergone previous radioactive iodine treament. Sixty patients were affected by Hashimoto’s thyroiditis as judged by the presence of serum anti-thyroglobulin and anti-thyroperoxidase antibodies and final histological diagnosis. Written informed consent was obtained from each patient; for patients below 18 yr of age the written informed consents were obtained by the patient’s parents. The study was approved by the ethical committee of the Policlinico Umberto I hospital of Rome.

**Figure 1:**
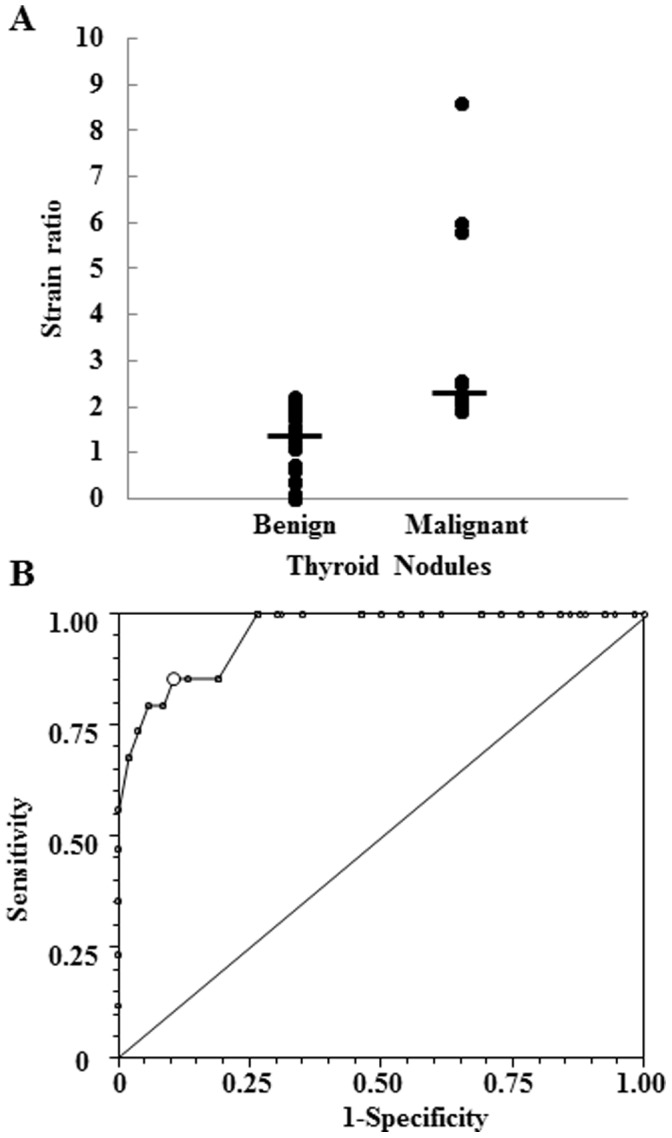
Distribution of the strain ratio in thy3 benign and malignant thyroid nodules (A) and Receiver Operating Characteristic (ROC) analysis (B) used to identify the optimum cut-off value of the strain ratio to discriminate between benign and malignant Thy3 thyroid nodules.

**Figure 2:**
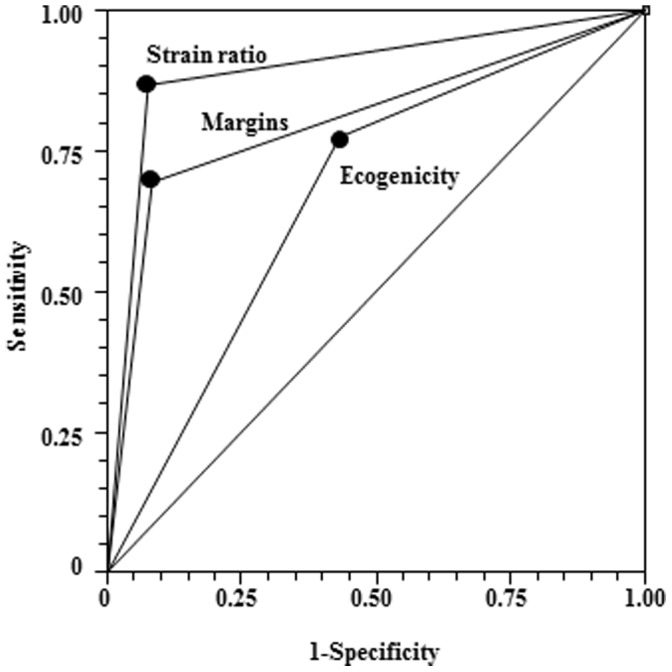
Comparison of the Receiver Operating Characteristic (ROC) curves of the strain ratio, irregular margin and hypoechogenicity/mixed nodules in the diagnosis of malignant Thy3 thyroid nodules.

### Ultasonography (US), Power Doppler US (PDUS) and Quantitative-US Elastography (Q-USE)

Before fine-needle aspiration cytology (FNAC) examination, nodules were examined by US, PDUS and Q-USE employing a Toshiba Aplio XVG, using a flat base 5–13 MHz probe, and for Q-USE the Elastography-Q software (Toshiba, Osaka, Japan).

**Table 1:** Diagnostic performance of different US parameters and Q-USE in the diagnosis of 140 cytologically indeterminate (Thy 3) thyroid follicular lesions.

	Malignant nodules	Benign nodules	Sensitivity	Specificity	NPV	PPV	Accuracy	OR	*p* value
**Ecogenicity**									
Ipoechoic/mixed	31	43	77.5	57.0	86.4	41.9	67.2	4.6	<0.001
Others	9	57	(61.5–89.2)	(46.7–66.9)	(75.7–93.6)	(30.5–53.9)	(59.1–75.4)	(2.0–10.4)	
**Microcalcifications**									
Present	20	36	50.0	64.0	76.2	35.7	57.0	1.8	0.127
Absent	20	64	(33.8–66.2)	(53.8–73.4)	(65.7–84.8)	(23.4–49.6)	(47.8–66.2)	(0.9–3.7)	
**Irregular Margins**									
Present	28	8	70.0	92.0	88.5	77.8	81.0	26.8	<0.001
Absent	12	92	(53.5–83.4)	(84.8–96.5)	(80.7–93.9)	(60.8–89.9)	(73.3–88.7)	(10.1–71.3)	
**Vascularization**									
Type 3	36	92	90.0	8.0	66.7	28.1	49.0	0.8	0.703
Others	4	8	(76.3–97.2)	(3.5–15.2)	(34.9–90.1)	(20.5–36.8)	(43.6–54.4)	(0.2–2.6)	
**Strain ratio**									
<2.05	5	92	87.5	92.0	94.8	81.4	89.8	80.5	<0.001
>2.05	35	8	(73.2–95.8)	(84.8–96.5)	(88.4–98.3)	(66.6–91.6)	(83.9–95.6)	(25.1–257.0)	

NPV, negative predictive value; PPV, positive predictive value; OR, odds ratio. In brackets the 95% confidence interval.

The US parameters evalauted included echogenicity (hypoechoic, hyper echoic, isoechoic or mixed), presence or not of irregular margins, diameters, presence or absence of microcalcifications (spot<2 mm). Following PDUS analysis, nodule blood flow was classified as: type 1, absent; type 2, presence of marked peripheral blood flow; type 3, presence of intralesional blood flow. Malignancy was suspected for nodules showing mixed or hypoechoic pattern, microcalcification, irregular margins and type 3 blood flow.

**Table 2:** Effects of nodule size on the diagnostic performance of different US parameters and Q-USE in the diagnosis of cytologically indeterminate (Thy 3) thyroid follicular lesions.

*Major diameter <1 cm (n = 32)*												
	Malignantnodules	Benignnodules	Sensitivity	Specificity	NPV	PPV	Accuracy		OR			*p* value
**Echogenicity**												
Ipoechoic/mixed	11	10	91.7	50.0	90.9	52.4	70.8		11.0			0.016
Others	1	10	(61.5–99.8)	(27.2–72.8)	(58.7–99.8)	(29.8–74.3)	(56.9–84.7)		(1.46-nd)			
**Microcalcifications**												
Present	8	8	66.7	60.0	75.0	50.0	63.3		3.0			0.144
Absent	4	12	(34.9–90.1)	(36.1–80.9)	(47.6–92.7)	(24.7–75.3)	(45.6–81.1)		(0.7–12.8)			
**Irregular margins**												
Present	12	0	100	100	100	100	100		nd			<0.001
Absent	0	20	(73.5–100)	(83.2–100)	(83.2–100)	(73.5–100)	(100–100)		nd			
**Strain ratio**												
<2.05	1	16	91.7	80.0	94.1	73.3	85.8		44.0			<0.001
>2.05	11	4	(61.5–99.8)	(56.3–94.3)	(71.3–99.9)	(44.9–92.2)	(73.7–98.0)		(5.2-nd)			
***Major diameter >1*** ** ***cm (n = 108)***												
**Echogenicity**												
Ipoechoic or mixed	20	33	71.4	58.8	85.5	37.7	65.1		3.6			0.006
Others	8	47	(51.3–86.8)	(47.2–69.6)	(73.3–93.5)	(24.8–52.1)	(55.0–75.2)		(1.4–8.9)			
**Microcalcifications**												
Present	12	28	42.9	65.0	76.5	30.0	53.9		1.4			0.459
Absent	16	52	(24.5–62.8)	(53.5–75.3)	(64.6–85.9)	(16.6–46.5)	(43.2–64.6)		(0.6–3.3)			
**Irregular margins**												
Present	16	8	57.1	90.0	85.7	66.7	73.6		12.0			<0.001
Absent	12	72	(37.2–75.5)	(81.2–95.6)	(76.4–92.4)	(44.7–84.4)	(63.7–83.5)		(4.3–33.6)			
**Strain ratio**												
<2	4	76	85.7	95.0	95.0	85.7	90.4		114.0			<0.001
>2	24	4	(67.9–96.0)	(87.7–98.6)	(87.7–98.6)	(67.3–96.0)	(83.3–97.4)		(27.3–476.0)			

NPV, negative predictive value; PPV, positive predictive value; OR, odds ratio. In brackets the 95% confidence interval. nd, non-determinable.

In the Q-USE, the technique allows compression of the target tissue with the same probe and the visualization, even if not in real time, of the dynamics of compression by recording a compression/time curve, in order to standardize measures on a sinusoid-shaped compression-decompression curve. Insufficient normal thyroid tissue around the target mass, which provides an adequate strain index, was considered an exclusion criterion. The ultrasound probe was placed gently on the thyroid in a longitudinal orientation with the patient in supine position. A series of compressions was performed; the compression dynamics was visualized and data were recorded if the dynamics met the requirements. Then, in off-line processing, we calculated strain images from radiofrequency data stored during US examination, by using the most symmetric dynamic [22]. Then, we evaluated the strain ratio, taking it in the last cycles. The strain ratio (normal tissue to lesion strain ratio) was calculated by dividing the strain value of the normal tissue at nearly the same height of the lesion by that of the nodule [22].

**Table 3:** Effects of thyroiditis on the diagnostic performance of different US parameters and Q-USE in the diagnosis of cytologically indeterminate (Thy 3) thyroid follicular lesions.

*Absence of thyroiditis (n = 80)*									
	Malignantnodules	Benignnodules	Sensitivity	Specificity	NPV	PPV	Accuracy	OR	*p* value
**Echogenicity**									
Ipoechoic/mixed	19	30	95.0	50.0	96.8	38.8	72.5	19.0	<0.001
Others	1	30	(75.1–99.9)	(36.8–63.2)	(83.3–99.9)	(25.2–53.8)	(64.5–80.5)	(3.0-nd)	
**Microcalcifications**									
Present	12	24	60.0	60.0	81.8	33.3	60.0	2.25	0.119
Absent	8	36	(36.1–80.9)	(46.5–72.4)	(67.3–91.8)	(18.6–51.0)	(47.3–72.7)	(0.8–6.2)	
**Irregular margins**									
Present	16	8	80.0	86.7	92.9	66.7	83.3	26.0	<0.001
Absent	4	52	(56.3–94.3)	(75.4–94.1)	(82.7–98.0)	(44.7–84.4)	(73.3–93.3)	(7.1–93.3)	
**Strain ratio**									
<2.05	1	54	95.0	90.0	98.2	76.0	92.5	171	<0.001
>2.05	19	6	(75.1–99.9)	(79.5–96.2)	(90.3–100)	(54.9–90.6)	(86.3–98.7)	(23.4-nd)	
***Presence of thyroiditis (n = 60)***									
**Echogenicity**									
Ipoechoic or mixed	12	13	60.0	67.5	77.1	48.0	63.8	3.1	0.042
Others	8	27	(36.1–80.9)	(50.9–81.4)	(59.9–89.6)	(27.8–68.7)	(50.5–77.0)	(1.0–9.3)	
**Microcalcifications**									
Present	8	12	40.0	70.0	70.0	40.0	55.0	1.6	0.439
Absent	12	28	(19.1–63.9)	(53.5–83.4)	(53.5–83.4)	(19.1–63.9)	(41.8–68.2)	(0.5–4.7)	
**Irregular margins**									
Present	12	0	60.0	100	83.3	100	80.0	nd	<0.001
Absent	8	40	(36.1–80.9)	(91.2–100)	(69.8–92.5)	(73.5–100)	(69.0–91.0)	nd	
**Strain ratio**									
<2.05	4	38	80.0	95.0	90.5	88.9	87.5	76.0	<0.001
>2.05	16	2	(56.3–94.3)	(83.1–99.4)	(77.4–97.3)	(65.3–98.6)	(77.9–97.1)	(13.5–412.0)	

NPV, negative predictive value; PPV, positive predictive value; OR, odds ratio. In brackets the 95% confidence interval. nd, non-determinable.

**Table 4:** COX regression analysis of different US parameters and Q-USE in the diagnosis of cytologically indeterminate (Thy 3) thyroid follicular lesions.

Variable	Odds ratio	95% confidence interval	*p* value
**Strain ratio**	74.22	(15.57–353.82)	<0.001
**Irregular margins**	17.95	(3.84–83.84)	<0.001
**Hypoechogen/mixed**	0.40	(0.08–2.10)	0.279
**Type 3 vascularization**	1.53	(0.07–33.84)	0.788
**Microcalcifications**	0.69	(0.17–2.79)	0.598

### Fine-needle Aspiration Cytology (FNAC) and Histopathology

All patients were instructed not to take aspirin or any other anticoagulants in the 5 days prior to biopsy. The scanning was performed with the patients in the supine position, and with the patients’ neck hyperextended. A 23- or 27-gauge needle, attached to 20 ml plastic syringes, was used to aspirate nodes under US assistance. All aspirates were smeared directly on glass slides and smeared with a second slide to spread the material across the surface for cytological examination. The slides were then either hair-dried or wet-fixed using the Bio-Fix (Bio-Optica, Milan, Italy). Hair-dried slides were stained with a May Grunwald-Giemsa solution, while the wet-fixed slides were stained with the Papanicolau solution.

Soon after thyroidectomy, the tissue was placed in 10% buffered formalin and paraffin embedded for histological diagnosis. Four μm tick sections were then prepared and stained with hematoxylin and eosin. All cytological and histopathological diagnoses were made by two expert pathologists (FN, VA), both blinded for the US, PDUS and Q-USE parameters. Cytological and histopathological diagnoses were defined according to widely recognized guidelines [18], [41].

### Statistical Analysis

Univariate analyses using the chi-squared test and multiple logistic regression analysis were performed to evaluate the relationship between categorical data and final histological outcome.

The sensitivity, specificity, positive predictive value, negative predictive value and diagnostic accuracy of each test, were calculated. Optimun cut-off value for strain ratio was calculated by Receiver operating characteristic (ROC) analysis. Areas under the ROC curve were compared using Bonferroni test. For each test, the results were considered statistically significant when the two-tailed p value was less than 0.05. All statistical calculations were performed using Stata version 8.0 (College Station, Texas, Stata Corporation, 2003).

## Results

Following surgery because of Thy3 cytological diagnosis, 40 nodules (28.6%) of the 140 analyzed were found to harbor a malignant lesion by histopathology. These included 34 papillary and 6 follicular thyroid carcinomas. The benign nodules included 70 hyperplastic nodules and 30 follicular adenomas.

As depicted in figure 1A, malignant Thy3 nodules showed a median strain ratio value of 2.2, which was significantly (p = 0.0008) higher than the 1.4 median value observed in the benign ones. Receiver operating characteristic (ROC) analysis demonstrated that the optimum strain ratio cut-off value for discriminating between benign and malignant lesions was 2.05 (see figure 1B). We then evaluated the diagnostic performance of the strain ratio against that of different US parameters. As shown in table 1, univariate analysis showed that among US and Q-USE parameters analyzed, hypoechogen or mixed nodules, the presence of irregular margin and strain ratio were significantly associated with malignancy, while microcalcifications and type 3 nodule vascularization did not. The Bonferroni test (see figure 2), used to compare the diagnostic accuracy of strain ratio, irregular margins, and hypoechogenicity showed that strain ratio (ROC area = 0.8975) and irregular margin (ROC area = 0.8100) had similar and not significantly different diagnostic accuracies (p = 0.0625). On the other hand, the diagnostic accuracy of hypoechogenicity (ROC area = 0.6725) was significantly lower than that of the strain ratio (p<0.0001) and irregular margins (p = 0.0092).

We then wondered whether tumor size and presence or absence of thyroiditis could affect the diagnostic accuracy of the above mentioned US and Q-USE parameters. In our study, we did not analyze nodule vascularization as only 12 out of the 140 nodules showed a vascularization type other than 3 and, when divided into 2 groups, the number was too low for statistical evaluation. As to the tumor size, the largest diameter in the 140 nodules analyzed showed a mean size of 18.4 mm ±10.66 mm (range 5–50 mm) with 32 having the largest diameter below 1 cm. As reported in table 2, the diagnostic accuracy of hypoechogen/mixed nodule, presence of irregular margin and strain ratio remained significantly associated with malignancy, regardless of the size of the nodules. However, while the diagnostic accuracy of hypoechogen/mixed nodule and the strain ratio did not vary significantly between the two groups, that of the irregular margins was significantly (p<0.0001) higher in nodules below 1 cm compared with that observed in nodules above 1 cm in diameter, where 100% accuracy was reached. In nodules below 1 cm, ROC analysis showed that irregular margins had a diagnostic performance statistically higher than that of hypoechogenicity (p = 0.0001) and strain ratio (p = 0.0045). On the other hand, in nodules above 1 cm in diameter, the strain ratio showed the best diagnostic accuracy which was significantly higher than that of hypoechogenicity (p = 0.0000) and irregular margin (p = 0.0011).

In table 3, the effects of thyroiditis on the diagnostic performance of the strain ratio and the different US parameters are showed. As shown in table 3, the diagnostic accuracy of hypoechogen/mixed nodule, the presence of irregular margins and strain ratio remained significantly associated with malignancy, regardless of the presence or absence of thyroiditis, and no significant differences between the two groups were observed.

Finally, multivariate logistic regression confirmed the significant association of Q-USE strain ratio and US irregular margins parameters with malignancy, but not that of hypoechogenicity (table 4).

## Discussion

To date, fine-needle aspiration cytology (FNAC) represents the main diagnostic tool for the evaluation of both palpable and non-palpable thyroid nodules. However, FNAC suffers from the major diagnostic limit represented by follicular lesions (Thy3), in which the cellular atypia encountered by cytopathologists are of indeterminate significance, thus preventing a distinction between benign and malignant lesions [16]. A number of different diagnostic approaches have been proposed in order to overcome the diagnostic limit of FNA [5]–[9]. However, all of them, including patient’s clinical characteristics, ultrasonography (US) parameters and the identification of genetic alterations in fine-needle aspiration material, turned out to be of limited clinical efficacy [5]–[9]. In recent years, a number of reports indicated the ultrasound elastography (USE) as a new diagnostic tool useful in the diagnosis of different human malignancies, including breast cancer [40]. Over the last few years, qualitative USE has been used by several investigators to differentiate benign from malignant thyroid nodules in unselected patients as well as in patients with indeterminate or non-diagnostic cytology (Thy3) [21]–[39]. Despite initial encouraging results, recent reports do not confirm the usefulness of USE in the pre-surgical selection of thyroid nodules and, in consequence, its utility has become a subject of considerable debate. In particular, the need for a quantitative (Q-USE) assessment of nodule stiffness to improve USE diagnostic accuracy has been invoked [38]. In this context, two recent reports on unselected patients demonstrated that Q-USE is more accurate than conventional US in the diagnosis of thyroid nodules and useful in patient’s presurgical selection [22], [26]. In this prospective study, the diagnostic accuracy of Q-USE and conventional US has been evaluated on a case study including 140 patients who underwent surgery following Thy3 cytological diagnosis. The final diagnosis showed that 28.6% of the above patients had a malignant lesion, a percentage which is in agreement with previously reported series [5], [21], [42], [43]. Q-USE confirmed previous studies and demonstrated that malignant nodules have a significantly higher stiffness compared to benign ones [23], [27]. Univariate analysis of the diagnostic performance of conventional US parameters, nodule vascularization and Q-USE showed that only hypoechogenicity, irregular margins and Q-USE were significantly associated with malignancy, with an overall accuracy of 67.2%, 81,0% and 89.8%, respectively. It is worth noting that the diagnostic accuracy of the irregular margins and that of Q-USE were not significantly different. Instead, their accuracies were significantly higher of that of hypoechonenicity. In agreement with previous studies, we found that Q-USE diagnostic accuracy, as well as that of hypoechogenicity and irregular margins, was not affected by nodule size or by the absence or presence of thyroiditis [26], [35], [42], [43]. It has to be mentioned, however, that in nodules below 1 cm the diagnostic performance of the irregular margin was significantly higher if compared to hypoechogenicity and strain ratio. Instead, in nodules above 1 cm, it was the strain ratio that showed a diagnostic accuracy statistically higher when compared to the irregular margins and hypoechogenicity. The above findings were further corroborated by the multivariate analysis demonstrating a statistically significant association of the strain ratio (Q-USE) and irregular margins with thyroid nodule’s malignancy, with odds ratio of 74 and 18, respectively.

In conclusion, to the best of our knowledge, this represents the first study assessing the diagnostic utility of Q-elastography in the differential diagnosis of thyroid nodules with indeterminate cytology. The high sensitivity, specificity, negative and positive predictive values of Q-USE here reported, if confirmed, could be of major clinical utility in the presurgical selection of patients with indeterminate cytology.
